# What does it mean if a patient is positive for anti-Jo-1 in routine hospital practice? A retrospective nested case-control study

**DOI:** 10.12688/f1000research.14834.1

**Published:** 2018-06-04

**Authors:** Paresh Jobanputra, Feryal Malick, Emma Derrett-Smith, Tim Plant, Alex Richter

**Affiliations:** 1 Department of Rheumatology, Queen Elizabeth Hospital, Birmingham, Birmingham, B15 2TH, UK; 2 Department of Rheumatology, Epsom and St Helier University Hospitals NHS Trust, Carshalton, Surrey, SM15 1AA, UK; 3 Department of Clinical Immunology, School of Medicine, University of Birmingham, Birmingham, B15 2TT, UK

**Keywords:** Jo-1, anti-histidyl-tRNA synthetase, synthetase syndrome, interstitial lung disease, myositis, connective tissue disease, over diagnosis

## Abstract

**Background:**  It is widely believed that patients bearing auto-antibodies to histidyl tRNA synthetase (anti-Jo-1) very likely have a connective tissue disease including myositis and interstitial lung disease.  The value of positive tests in low disease prevalence settings such as those tested in routine care is unknown.  We sought to determine the value of anti-Jo-1 auto-antibodies in routine practice.

**Methods**: Our study was a nested case control study within a retrospective cohort of all patients tested for anti-ENA our hospital, from any hospital department, between January 2013 and December 2014.  Data was extracted from electronic records of anti-Jo-1 positive patients and randomly selected ENA negative patients (ratio of 1:2), allowing for a minimum follow up of at least 12 months after first testing.

**Results:** 4009 samples (3581 patients) were tested.  Anti-ENA was positive in 616 (17.2%) patients, 40 (1.1%) were anti-Jo-1 positive. Repeat ENA testing was done for 350/3581 (9.8%) patients (428 of 4009 (10.7%) samples) and in 7/40 (17.5%) of anti-Jo-1 positive patients. The median interval between the first and second request was 124 days (inter-quartile range 233 days).  The frequencies of interstitial lung disease (ILD), myositis and Raynaud’s were comparable for anti-Jo-1 positive patients (n=40) and 80 randomly selected ENA negative controls.  Positive tests led to additional diagnostic testing in the absence of clinical disease.  Sensitivity of Jo-1 for ILD was 50% (CI 19-81%), specificity 68% (CI 59-77%), positive predictive value 12.5% (CI 4 to 27%) and negative predictive value 93.8% (CI 86-98%). Of 10 (25%) patients with high anti-Jo1 levels, 3 had ILD, one myositis and two a malignancy (disseminated melanoma and CML).

**Conclusion:** Anti-Jo-1 is uncommon in a heterogenous hospital population and is only weakly predictive for ILD.  Repeated test requests were common and potentially unnecessary indicating that controls over repeat requests could yield significant cost savings.

## Introduction

Diagnosis of a connective tissue disease (CTD) may be considered in many clinical situations because of the diverse clinical manifestations of these diseases. Diagnosing a specific condition may be facilitated by a variety of immunological tests, including auto-antibodies to anti-nuclear antibodies (ANA) or extractable nuclear antigens (ENA) ^1, 2^. Anti-ENA antibodies consist of a panel including auto-antibodies associated with systemic lupus erythematosus, scleroderma, mixed connective tissue disease, myositis and Sjogren’s syndrome.

Data from the manufacturers of anti-ENA testing kits indicate that the diagnostic precision of anti-Jo-1 (sensitivity and specificity) exceeds 95% ^3^. The sensitivity and specificity of a test does not tell us about the probability of disease. The sensitivity and specificity data of many diagnostic tests are calculated from case-control studies, in which test positivity is compared in patients with a known connective tissue disease with healthy controls or controls with other diseases. These methods greatly over-estimate the positive predictive value (PPV) of a diagnostic test, because disease prevalence is increased in the assembled population by the methods used for calculation ^4^. Clinicians find diagnostic test accuracy data confusing and tend to over-estimate the probability of disease following a positive test result ^5^. Disease prevalence in populations tested for anti-ENA in routine care is rarely known because testing may be requested and performed for any patient and without constraints in most hospitals. Thus, the prevalence of relevant diseases is likely to be considerably lower. Positive test results in general hospital practice may thus lead to over-diagnosis, especially in sick hospital patients with multiple morbidities, and may lead to additional expensive testing, patient and clinician anxiety and overtreatment.

In this study we focus on anti-Jo-1 (histidyl-tRNA synthetase). This autoantibody is associated with inflammatory muscle diseases and interstitial lung diseases (ILD). We retrospectively identified a cohort of patients tested for the panel of anti-ENA autoantibodies in our hospital. Using a nested case-control design we identified patients who tested positive for anti-Jo-1 and compared them with controls, from the same source population, who tested negative for anti-Jo-1. We sought to determine the accuracy and value of this test for hospital medical practice.

## Methods

### Patients and controls

The patient population (P) included in the present study was those in whom an ENA test was requested (I), from any department in Queen Elizabeth Hospital Birmingham (Birmingham, UK) over 2 years, between January 2013 and December 2014. The patient population was identified from records in the Immunology laboratory. This hospital is a large regional teaching hospital and referral center. Patients who were positive for anti-Jo-1 were the population of interest and randomly selected patients negative for anti-Jo-1 served as controls (C). The outcome of interest was a clinical diagnosis of myositis or ILD (O), determined by a clinical diagnosis of these conditions within patient medical records.

ENA tests are conducted sequentially by the University of Birmingham Immunology Laboratory. First, samples are screened for ENA auto-antibodies using the Quanta Lite® ENA 6 ELISA (Inova Diagnostics). Screen-negative patients are not tested further. Screen-positive patients have levels of auto-antibodies to ENA sub-types quantified by Quanta Lite products for each of the 6 ENAs screened: anti-Sjögren’s Syndrome A antigen (SSA); anti-Sjögren’s Syndrome B antigen (SSB); anti-Scl-70 antigen (also known as DNA-topoisomerase-1); anti-ribonucleoprotein (nRNP; also known as U1RNP), a small nuclear ribonucleoprotein; anti-Smith (Sm) antigen, another small nuclear ribonucleoprotein; and anti-Jo 1 (histidyl-tRNA synthetase). These assays are semi-quantitative, thus numerical values are available for each of the ENA subtypes if a sample proves to be ENA-screen-positive and are then tested in detail. For anti-Jo-1, levels >20 AU/ml are deemed to be positive, according to manufacturer instructions.

Stored serum samples were available for some anti-Jo-1-positive patients. We retrospectively re-tested these samples on a second occasion with the same Quanta Lite® platform to confirm anti-Jo-1 positivity and compared levels with the original test result. The first sample tested, during the study period, was designated as the test of interest.

If patients were tested for ENA on more than one occasion between January 2013 and December 2014, the numbers of repeat tests per patient were counted and the interval between the first and subsequent tests during this 2-year study period calculated. We compared levels of anti-Jo-1 for patients who were tested on a second occasion to assess the stability of anti-Jo-1 values. This was only possible in samples that were ENA-screen-positive on both occasions, since screen negative samples are not tested with semi-quantitative measurements, as per our laboratory protocol.

Clinical and radiographic data from positive patients was extracted from hospital electronic records. We ensured that a minimum of 12-month clinical follow up was available after the first ENA test. This allowed time for any planned clinical and diagnostic evaluations to be completed and allowed time for the evolution of the clinical picture. A random sample of ENA-screen-negative patients served as controls, in a ratio of 2 controls for each ENA positive patient. Control patients were selected using the random number generator in Microsoft Excel.

### Ethics

Our study was registered with our hospital governance department (registration number CARMS-12140). Ethical approval was judged not necessary based on definitions provided by the NHS Health Research Authority ^6^.

### Statistics

Cases (anti-Jo-1-positive patients) were identified from the population of anti-ENA-tested patients and controls (anti-Jo-1-negative patients) selected randomly from that same population. Thus, our study was a nested case-control study ^7^. Supplementary File 1 contains a completed STARD checklist; Supplementary File 2 contains a completed STROBE checklist. We decided from the outset to include two controls for each case. Manufacturer data for sensitivity and specificity for the Quanta Lite product indicate that their anti-Jo-1 test is more than 99% specific and around 14% sensitive for polymyositis, yielding a positive likelihood ratio of 14. We assumed that these data hold true for our population and that each positive patient had disease (myositis and/or ILD) and that around 5% of controls had disease. Based on these assumptions and by substituting the likelihood ratio for the odds ratio, we estimated that around 18 cases and 54 controls would be needed to provide a power of 90% and alpha risk of 5% ^8^.

Statistical calculations were performed using Microsoft Excel 365. Descriptive statistics were used to compare key characteristics between patients and controls. Differences in proportions were compared using Fisher’s exact test. Where repeated testing was done for the same ENA positive patient, the result from the test of interest (first test during study period) was compared with the next available ENA test. This was done to evaluate test stability, using a Bland–Altmann plot. The diagnostic utility of anti-Jo-1 for a diagnosis of ILD and/or myositis, based on clinical diagnosis in patient records, was assessed by calculating sensitivity, specificity, positive predictive value (PPV) and negative predictive value (NPV).

## Results

**Figure 1.**
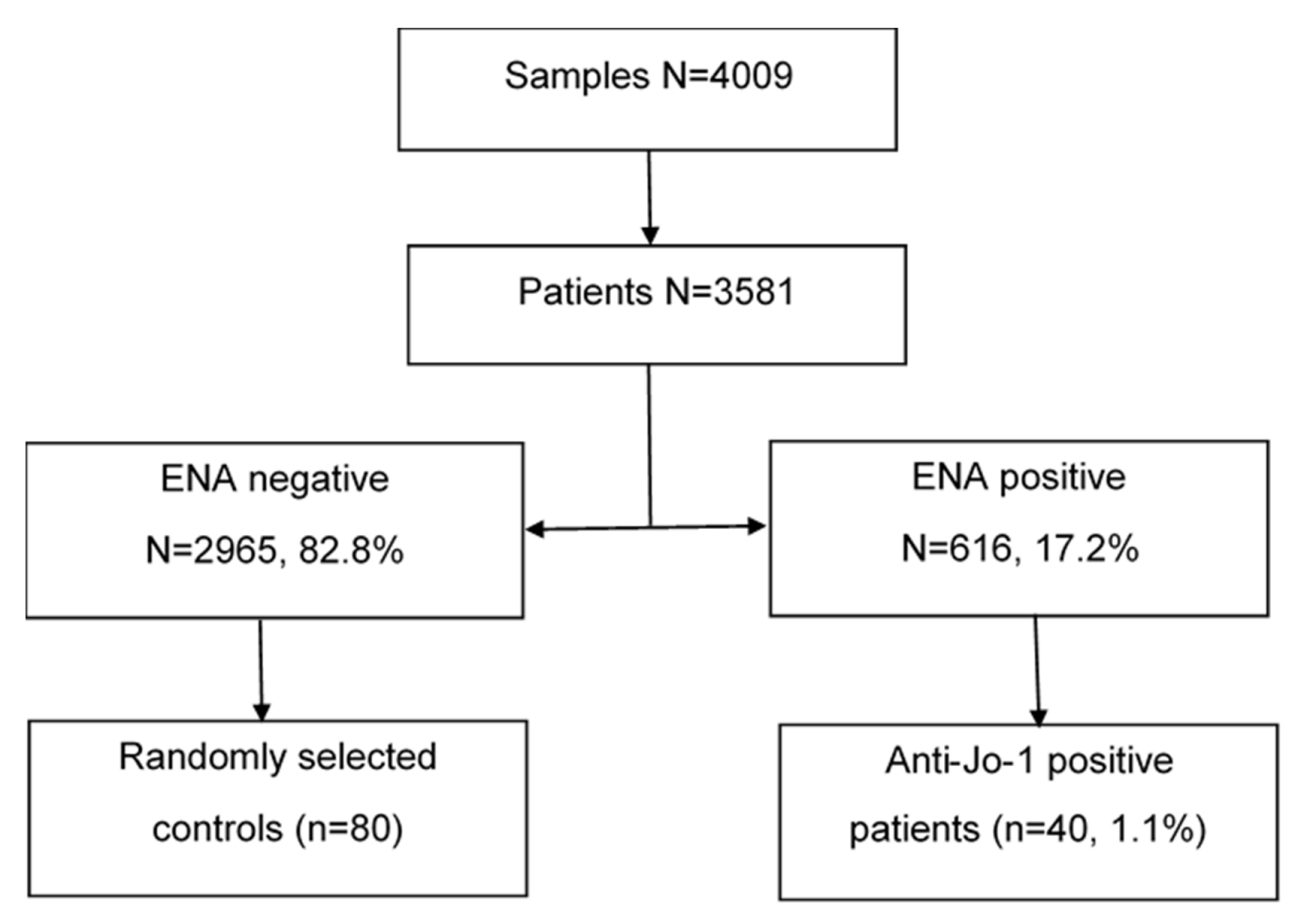
Study flow diagram.

A total of 4014 samples from 3584 patients were tested for ENA during the 2-year study period. There were 3 patients excluded because their samples originated from outside our hospital, leaving 4009 samples and 3581 patients. The first sample tested chronologically was designated the test of interest. Positive ENA tests occurred in 616 (17.2%) patients, 2965 (82.8%) patients were negative. The study flow diagram is shown in Figure 1. The frequency of positives at levels of ≥20 and ≥40 units are shown in Table 1. SSA, SSB, combined SSA and SSB, including patients with high concentrations of auto-antibodies, were commonly found in this population.

**Table 1.** Frequency of extractable nuclear antigen (ENA) sub-types in the ENA-screen-positive population (n=616). Other combinations of ENA serotypes occurred in fewer than 10 patients for each of the combinations not shown here.

ENA sub-type ^1^	Positive (≥20 units)	Positive (≥40 units)
SSA	326 (9.1%)	277 (7.7%)
SSB	165 (4.6%)	128 (3.6%)
Both SSA and SSB	158 (4.4%)	121 (3.4%)
RNP	127 (3.5%)	92 (2.6%)
Sm	58 (1.6%)	35 (1.0%)
Both RNP and Sm	56 (1.6%)	33 (0.9%)
Jo-1	40 (1.1%)	10 (0.3%)
Scl-70	29 (0.8%)	13 (0.4%)
Both RNP and SSB	18 (0.5%)	6 (0.2%)

^1^Arranged by frequency.

We studied the anti-Jo-1 positive patients (40, 1.1%) in detail and selected 80 ENA negative patients, randomly, as controls. Key clinical characteristics of these two populations are shown in Table 2.

**Table 2.** Demographic and clinical characteristics of anti-Jo-1-positive patients versus extractable nuclear antigen (ENA)/anti-Jo-1 negative controls.

Variable	Jo-1 positive (n=40)	Controls (n=80)	P value *
Age, mean years (range)	53 (19–86)	52 (17–87)	
Sex (% female)	70%	79%	0.37
Dead	13%	4%	0.12
Current or previous malignancy	10%	10%	1.0
Raynauds	17.5%	6.3%	0.10
Inflammatory arthritis	20%	19%	1.0
Clinical myositis diagnosis	5%	1.3%	0.26
CPK >1000 U/l	5%	1.3%	0.26
Interstitial lung disease	12.5%	6%	0.30
CT chest done during study period, n/N	17/40	20/80	0.06
ANA ≥1:100, n/N	18/38 (47.4%)	22/79 (27.8%)	0.06
RF, n/N	8/25 (32%)	12/44 (27.3%)	0.78
CCP, n/N	0/19 (0%)	3/33 (9.1%)	0.54
Anti-dsDNA (Crithidia +ve)	7.5%	1.3%	0.11
Scl70	7.5%	0%	**
SSA/Ro	10%	0%	**
SSB/La	10%	0%	**
RNP	10%	0%	**

*Fisher’s exact test, two tailed. **Statistical analyses were not done for these comparisons, controls were negative for ENA antibodies, by definition.

A total of 350 patients (9.8%; 428 samples or 10.7% of all samples) were tested on more than one occasion, 55 patients were tested on 3 or more occasions and 10 patients were tested on 4 or more occasions. Of the 40 (1.1%) anti-Jo-1-positive samples, 7 had the test done on more than one occasion. The median interval between the first and second test request for 350 patients was 124 days (interquartile range, 233 days; mean 168 days).

**Figure 2.**
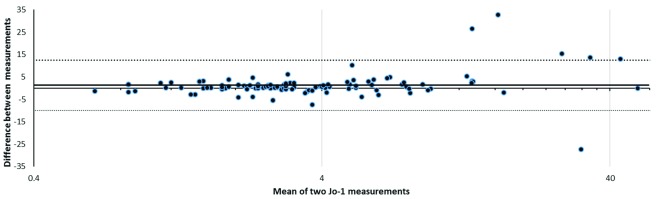
Bland–Altman plot of extractable nuclear antigen-screen-positive patients with at least two Jo-1 measurements (n=109 patients). Jo-1 is considered positive if >20 AU/ml. The bold horizontal line shows the bias and dotted lines show upper and lower limits of agreement. Narrow limits of agreement are shown, but note that agreement appears to deteriorate at higher values of Jo-1. The bias or mean difference value (bold horizontal line) is small.

The stability of Jo-1 values between the first and second test, where a second test was requested later and where numerical data (ENA screen positive) were available, are shown in Figure 2. This illustrates that values remain stable between repeat tests over a median of 124 days, but that stability appears to lessen as anti-Jo-1 values rise. The likelihood of tests being repeated was greater in ENA-positive patients (18.8%) than in ENA-negative patients (7.8%), p<0.0001, Fisher’s exact test.

**Figure 3.**
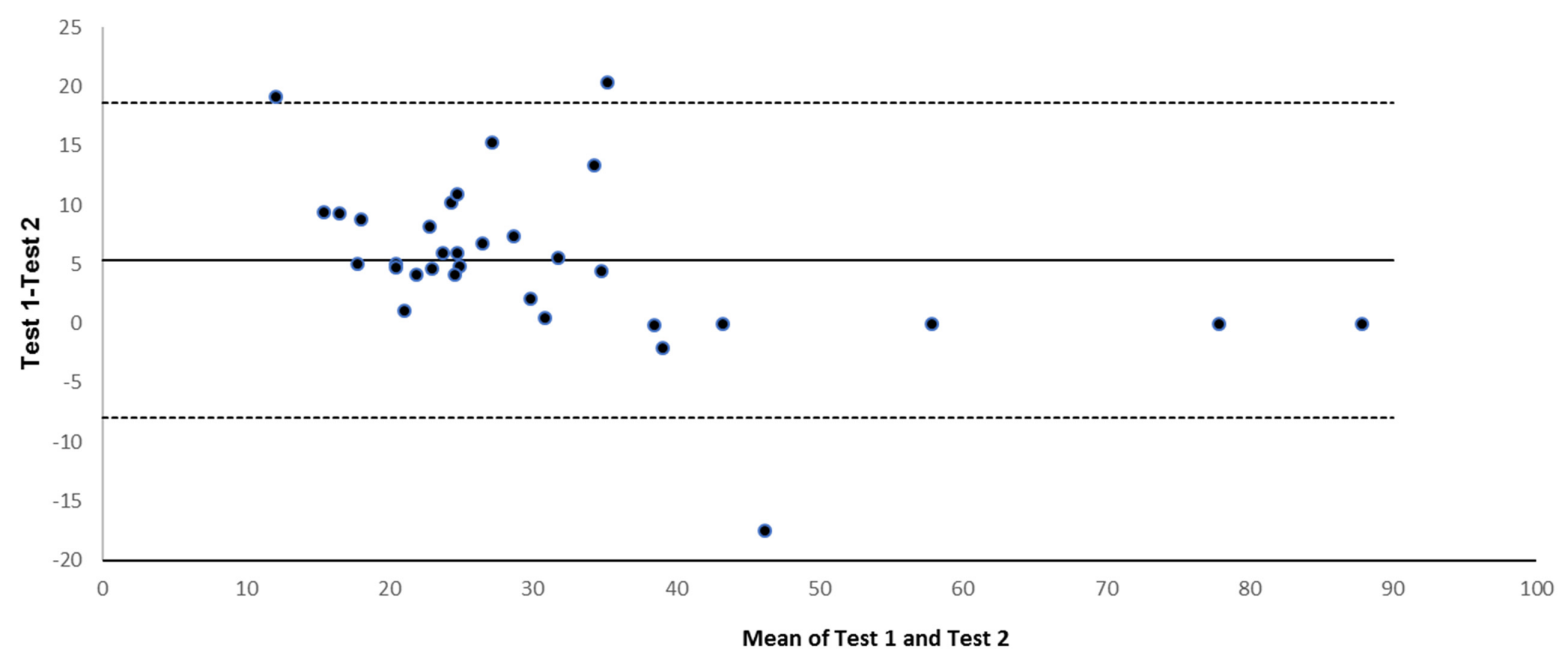
Bland–Altman plot of anti-Jo-1-positive (>20 AU/ml when first tested) patients compared with re-test result on stored serum re-tested using Quanta Lite assay for anti-Jo-1 (n=33). The bold horizontal line shows the bias and dotted lines show upper and lower limits of agreement.

Finally, using available stored samples from Jo-1-positive patients (n=33), we checked whether repeat measurement on the same sample using the same Quanta Lite® platform yielded consistent value ( Figure 3). In three cases the first test result was reported as >50 U/ml (per laboratory practice at that time), whereas on retesting, more precise values were reported. For analysis, we assumed, in these strongly positive cases, that the original test results were identical to the more precise values reported on repeat testing. The degree of agreement between these repeat measures on the same sample is visualized using a Bland–Altman plot ( Figure 3), the bias (average difference between repeat measures) was 5 AU/ml (95% CI, −8.0–18.7). We were concerned that auto-antibody levels in stored samples would have degraded over time; however, results from stored samples were not consistently lower or higher than the index test result ( Figure 3).

### Clinical characteristics of anti-Jo-1 positive patients

Clinical and demographic data all from 40 anti-Jo-1-positive patients was compared with 80 controls (ENA negative) ( Table 2). The mean follow up time from the first anti-ENA test to review of medical records was 2.5 years (range, 1.6–3.5 years). When comparing the frequency of ILD, myositis and Raynaud’s, no statistically significant differences were found. The sensitivity and specificity of Jo-1 for ILD, as the key feature of ‘anti-synthetase syndrome’, were 50% (95% CI, 19–81%) and 68% (95% CI, 59–77%), respectively; the PPV was 12.5% (95% CI, 4–27%) and NPV was 93.8% (95% CI, 86–98%). Very few patients had other described features of anti-synthetase syndrome or the full spectrum of this disorder to calculate diagnostic accuracy data. There are no gold standards or widely accepted classification criteria for anti-synthetase syndrome ^9^. Patients with anti-Jo-1 had a very wide range of co-morbidities ( Table 3).

**Table 3.** Morbidity associated with positive anti-Jo-1 (n=40).

Conditions	Patients, n (%)
Pulmonary disease	
Interstitial lung disease	5 (12.5%)
Chronic obstructive pulmonary disease or asthma	6 (15%)
Other: pneumonia (2); alpha-1 anti-trypsin deficiency (1); previous hemo-pneumothorax (1);	5 (12.5%)
Rheumatic Disease	
Polymyositis / dermatomyositis	2 (5%)
Polymyalgia rheumatica/Rheumatoid arthritis or unspecified inflammatory arthritis	4 (10%)
Sjogren’s syndrome	3 (7.5%)
Systemic lupus erythematosus	3 (7.5%)
Discoid lupus	2 (5%)
Scleroderma (limited or systemic)	1 (2.5%)
Raynauds	1 (2.5%)
Osteoarthritis (including spine)	8 (20%)
Others: Myalgia / arthralgia (4); fibromyalgia (2); gout (1); polyangiitis (1);	8 (20%)
Malignancy (current or previous)	
Cervical cancer (1); metastatic malignant melanoma (1); chronic myeloid leukaemia (1); breast cancer (1)	4 (10%)
Miscellaneous	
Cardiovascular (including ischaemia, atrial fibrillation, hypertension)	11 (27.5%)
Hepatobiliary: liver transplant (1), hepatitis or unspecified liver disease (6), autoimmune hepatitis (1); primary biliary cirrhosis (1))	9 (22.5%)
Renal: acute kidney injury (2); chronic kidney disease (3); lupus nephritis (2); urinary tract infection (1)	8 (20%)
Skin disorders: lichen planus (1); urticaria (1); eczema (3); alopecia (1)	6 (15%)
Neurological & psychiatric (inc. strokes and neuropathy): depression (4);	6 (15%)
Endocrine disorders: type II diabetes (2); hypothyroid (3)	5 (12.5%)
Gastrointestinal: celiac disease (1); ulcerative colitis (1); irritable bowel syndrome (1)	3 (7.5%)
Others: Benign prostatic hyperplasia (1); mesenteric vein thrombosis (1); Angioedema (1)	3 (7.5%)

In a sub-group of patients with the highest anti-Jo-1 titers (≥40 AU/ml; 10/40 patients), 9 had a chest CT scan at some stage of their illness: one patient with metastatic melanoma and lung involvement including evidence of ILD and raised muscle enzymes was given a diagnosis of anti-synthetase syndrome; two other patients had ILD and normal muscle enzymes, one of these patients had systemic lupus erythematosus and lupus nephritis; one patient had chronic myeloid leukemia, one congestive cardiac failure and polymyalgia rheumatica, one Sjögren’s syndrome, two osteoarthritis (one with emphysema), and two with hepatitis (one labelled as autoimmune). Records indicated that two patients had chest CT scans primarily because of a positive anti-Jo-1, not because respiratory disease was found. In both these cases chest scans were normal. Only one of the anti-Jo-1-positive patients had a recorded creatine phosphokinase (CPK) level >1000 units/liter (the patient with metastatic melanoma)) and another had a diagnosis of myositis with a normal CPK level. Muscle biopsies were not done in any of these patients.

Complete data for all participants regarding disease status and results of extractable nuclear antigen (ENA) testing Data are presented as ENA-negative controls, Jo-1-positive patients and data pooled from all patients. Click here for additional data file. Copyright: © 2018 Jobanputra P et al. 2018 Data associated with the article are available under the terms of the Creative Commons Zero "No rights reserved" data waiver (CC0 1.0 Public domain dedication).

## Discussion

Studies such as this one, which report on the diagnostic value of a test in routine care and where patients have a wide variety of clinical features, are uncommon. Often, academic estimations of the diagnostic value of a test are assessed by comparing positivity in patients with confirmed disease against those with no disease or another disease. This approach increases the prevalence of diseased subjects in the population used to make calculations of diagnostic accuracy. Diagnostic sensitivity and specificity of a test may vary with disease prevalence and with disease spectrum, for example disease severity and the presence of co-morbidity ^4, 11^. Thus, a test may perform better where selected cohorts of patients with known disease and more severe disease are included, as seems likely to occur in cohorts of patients from referral centers, where much of the data on the relationship between Jo-1 and disease originate. The true value of a test only becomes apparent when it is used in usual clinical settings where diagnosis may be uncertain, other serious illnesses present, symptoms are unexplained or where there is multi-morbidity.

The PPV of anti-Jo-1 for ILD in our study was 12.5%. This figure is well below that produced by another study, in which anti-Jo-1 was concluded to be highly specific for autoimmune myositis but not thought to be sensitive ^12^. Our patients were tested using a commercial ELISA for anti-ENA and anti-Jo-1. These assays have been compared with other methods and other manufacturers’ immunoassays, and are believed to be reliable ^13^. Nevertheless, anti-Jo-1 detected by ELISA may lack specificity ^13, 14^. Older techniques are believed to be more specific and use native antigens in soluble form, whereas ELISAs and other assays, such as addressable laser bead immunoassays, use antigen-coated surfaces ^9^. The use of a solid phase in ELISA could cause conformational changes to antigens or antigen denaturation, influencing test performance.

A plea for greater clinical correlation and consideration before introducing new assays, particularly in the context of local test usage, was made over a decade ago ^15^. Indeed, the UK National External Quality Assessment Service (UKNEQAS) recommends that a less sensitive or more specific test, such as indirect immunofluorescence (IIF), should be used to screen for samples prior to testing by more sensitive methods ^15^. However, UKNEQAS reports considerable differences in the interpretation of IIF. Regarding ENA testing, UKNEQAS note: “a more worrying observation is the continuing complete lack of homogeneity and agreement of reported ENA specificities across the various manufacturers for almost any ENA type” ^16^.

Anti-Jo-1 is grouped with autoantibodies believed to be specific for myositis ^17^, and occurs very rarely in normal populations ^18^. Around 22% of patients with polymyositis diagnosed in specialist centers had anti-Jo-1 ^19^ In addition when clinical data was reviewed for anti-Jo-1 positive patients, identified from a laboratory database, 70% had ILD ^20^. This compares with 12.5% ILD in our anti-Jo-1-positive cohort. An association of myositis and ILD has led to coining of the term ‘anti-synthetase syndrome’. The notion of a unique syndrome attributable to the presence of auto-antibodies to synthetases has been challenged by a review of cohort studies ^9^; our data add to this doubt. We showed that 17% of our population screened positive for anti-ENAs, most commonly SSA and or SSB. Anecdotally, we know that these positive tests result in referral from primary care to secondary care. Anti-Jo-1 occurred in 1.1% of our patients, including many without a recognized CTD, myositis or ILD.

Our study has notable limitations. First, its retrospective design means that systematic data collection, for example specialist review and specific steps to determine the presence of Raynaud’s, ‘mechanics hands’, myositis or ILD, were not planned from the outset. Second, patients found to be positive for anti-Jo-1 could have been investigated more diligently than those found to be negative: an example of work up or verification bias. Thus, there could have been a greater propensity to diagnose ILD and use diagnostic tests such as chest CT scans. We identified two patients with high titer anti-Jo-1 who had such scans motivated purely by a positive anti-Jo-1 test.

We relied on routinely collected clinical data recorded in hospital electronic records and depended on clinical diagnoses rather than applying disease classification criteria. We did not access primary care records and may also have missed data if patients had been seen at other hospitals or in private health clinics. However, a strength of our study was the inclusion of a large cohort of patients seen in a variety of clinical settings. A retrospective design allowed our study to be completed with limited resources and in a timely manner. The inclusion of a heterogeneous population provides more reliable estimates of the value of anti-ENA and anti-Jo-1 in hospitals, the setting where these tests are likely to be ordered. We judged that a follow-up period of 1 year after the first anti-ENA test was done, provided sufficient time for key clinical features to emerge, especially ILD. We recognize, though, that auto-antibodies may precede CTD by many years and acknowledge that more prolonged follow-up may have given added value.

We show that anti-Jo-1 occurs in patients with varied illnesses, including cancer and other autoimmune diseases, raising the possibility that seriously ill patients with multiple morbidities may develop auto-antibodies at significant levels without relevant auto-immune diseases, but perhaps because of tissue damage. Auto-antibodies found in myositis have been classified into those believed to be specific for myositis (for example anti-Jo-1) and those that are associated with myositis. This distinction implies a special role for certain autoantibodies in disease pathogenesis. For anti-Jo-1, this view may be justified since this autoantibody can cause lung and muscle disease in animal models ^21^. Yet, our data suggest that caution is necessary in according anti-Jo-1 a prominent role in disease pathogenesis. Recent research indicates that histidyl-tRNA synthetases have a role beyond protein synthesis, such as maintenance of immune homeostasis and stimulating immunological responses ^22, 23^. This suggests that much more needs to be learnt about the potential immunopathogenic role of anti-Jo-1 and other anti-synthetases.

The development, evaluation and dissemination of diagnostic tests is not standardized. Additionally, methods of test evaluation are often sub-optimal ^24^. Test usage may become widespread and clinicians may not fully appreciate the limitations of a test in practice. A test believed to be highly specific for a disease owing to published or manufacturer data may lead to additional confirmatory diagnostic testing, including invasive testing, with the risk of over-diagnosis and patient harm.

Of the anti-synthetase auto-antibodies, only anti-Jo-1 is tested routinely. A plea for wider serological testing, especially in patients with ILD, was made recently ^25^. It is believed that patients with ILD who have auto-antibodies other than anti-Jo-1 have a worse prognosis. However, we believe that robust evaluations of the precision of newer auto-antibodies for diagnosis and determining prognosis, in heterogeneous populations likely to be tested in secondary care, are necessary before wider dissemination.

We found that 10.7% (428) of the samples tested were repeat requests for anti-ENA, in some cases on multiple occasions. The ‘Choosing Wisely’ initiative, supported by the American College for Rheumatology, recommends restraint and careful patient selection when first testing for ANA. Testing for anti-ENA is not recommended when the ANA is negative ^26^. It appears, therefore, that widespread inappropriate laboratory testing is occurring. Determining the appropriateness of testing, however, is problematic ^27^, but examples of good practice, at least for restricting repeated test requests, have been described by the UK Academy of Medical Colleges ^28^. We did not attempt to ascertain reasons for repeated requests, but such testing may be done where there is diagnostic uncertainty, borderline results or simply because of inadequate review of available data or not knowing that samples had recently been submitted. For example, we show that 17.3% of anti-ENA repeat requests were made within 7 days of the first request.

Restricting test requests or ‘gating’ has been implemented successfully for anti-neutrophil cytoplasm antibody (ANCA) in an English hospital ^29^, though such a policy requires clinician and laboratory staff time, which may mean that such a strategy is not cost effective. However, a policy to decline repeat requests within specified time periods could be implemented more readily and could be automated. Our hospital is in the process of implementing this for selected immuno-diagnostic tests.

Limited data suggests that anti-Jo-1 levels reflect disease activity ^30^. Our data shows that anti-Jo-1 levels measured by ELISA vary at higher concentrations when re-checked at a later point ( Figure 2). We also noted variation in test results when the same positive sample was re-tested ( Figure 3) after storage for many months.

In summary, we show that many hospital patients negative for ANA are tested for anti-ENA, often repeatedly. We show that positivity for anti-Jo-1 is uncommon and that positive results are poorly predictive for interstitial lung disease and clinically diagnosed autoimmune myositis.

## Data availability

The data referenced by this article are under copyright with the following copyright statement: Copyright: © 2018 Jobanputra P et al.

Data associated with the article are available under the terms of the Creative Commons Zero "No rights reserved" data waiver (CC0 1.0 Public domain dedication). 

**Dataset 1. Complete data for all participants regarding disease status and results of extractable nuclear antigen (ENA) testing.** Data are presented as ENA-negative controls, Jo-1-positive patients and data pooled from all patients. DOI: 10.5256/f1000research.14834.d204254 ^10^.

## Supplementary material

**Supplementary File 1. Completed STARD checklist.**

**Supplementary File 2. Completed STROBE checklist.**
