# Supplementary material for: What does it mean if a patient is positive for anti-Jo-1 in routine hospital practice? A retrospective nested case-control study
Source: F1000Res. 2018 Jun 4;7:698. [Version 1] doi: 10.12688/f1000research.14834.1 (PMC6058461; doi:10.12688/f1000research.14834.1)
Supplement: Peer review history

# Peer review: Referee response for version 1

**DOI:** 10.5256/f1000research.16147.r34692

**Published:** 2018-07-23

**Authors:** Stephen Adelstein, MaiAnh Nguyen

**Affiliations:** 1 Department of Clinical Immunology and Allergy, Royal Prince Alfred Hospital, Camperdown, NSW, Australia; 2 Faculty of Health and Medicine, Central Clinical School, University of Sydney, Sydney, NSW, Australia; 3 Department of Clinical Immunology, Concord Hospital, Sydney, NSW, Australia

The authors address an important question about the performance characteristics and clinical utility of autoantibody testing. They review the outcome of ENA testing requested in a general hospital where the tested patients apparently do not have a high probability for the disease associated with a specific ENA of interest, Jo-1. The paper is well-written and highlights many of the problems in the use and interpretation of autoantibody tests, particularly the use of an auto-antibody test to define a disease subgroup.

 Their findings are not surprising, concluding that there is no difference in the clinical associations with those patients in the cohort who were ENA negative compared to those who were ENA positive. It is well known that tests do not perform well when inappropriately selected in patients who do not have at least a reasonable pre-test probability of the disease in question. This paper illustrates this very well, but does not emphasize this aspect sufficiently in the discussion of the results.

 One other issue not discussed by the authors that likely impacts on their findings is the performance in laboratories of ENA tests as a group. In the authors laboratory a request for anti-ENA antibodies produces a request for a screen of antibodies to six ENAs, and if this test is positive, further tests are performed to identify to which of the six ENAs the antibodies bind. This is not an uncommon strategy in Immunology laboratories, but ignores the pre-test probability of diseases that prompted request for ENA testing. It is likely that many of the requests for ENA received in the laboratory were for diagnosis of other autoimmune diseases rather than myositis and ILD and that has clinicians had a choice of which ENA antibody to request they may not have chosen anti-Jo-1 antibodies as the clinical pre-test probability for requesting this ENA was low. The test was, nevertheless, performed because of the testing algorithm in operation in the laboratory. This scenario would no doubt affect the calculated positive predictive value in the paper.

 It would, therefore, be misleading from the information provided in this study, to conclude that anti-Jo 1 antibodies do not perform well when requested in the appropriate circumstances, that is, in individuals who have evidence of myositis and/or interstitial lung disease. In these patients a positive result for Jo-1 antibodies may be helpful in selecting appropriate treatment and in prognostication.

 This study highlights that anti-Jo-1 positivity, in the absence of clear clinical features, has a very low predictive value. As mentioned by the authors, a longer follow up period may be useful to determine whether there is any delayed predictive value of anti-Jo-1, like findings reported with anti-CCP positivity and rheumatoid arthritis.

 Although a much larger study would be required, it would have been interesting to correlate the authors’ findings with the rate of anti-Jo-1 positivity in patients already diagnosed with interstitial lung disease, at the same hospital.

We have read this submission. We believe that we have an appropriate level of expertise to confirm that it is of an acceptable scientific standard.

# Peer review: Referee response for version 1

**DOI:** 10.5256/f1000research.16147.r34691

**Published:** 2018-06-13

**Authors:** Robert Cooper

**Affiliations:** 1 MRC-Arthritis Research UK Centre for Integrated research into Musculoskeletal Ageing, University of Liverpool, Liverpool, UK

This is a well constructed piece of research which examines the diagnostic value (sensitivity and selectivity) of anti-Jo-1 positivity when detected by standard ENA testing in a large teaching hospital. The results clearly show that Jo-1 positivity is not particularly predictive of myositis or ILD, as many other non-CTD conditions can also be associated with a positive result. This is rather surprising, but the authors explain why this outcome. The reviewer has no negative comments to make about this article, but is not a statistical expert, so the view of a statistician may be warranted.

I have read this submission. I believe that I have an appropriate level of expertise to confirm that it is of an acceptable scientific standard.
